# Socio-Economic Status Differences in Changing Affordability of Tobacco Products from 2011–2012 to 2018–2019 in India

**DOI:** 10.1093/ntr/ntac230

**Published:** 2022-10-04

**Authors:** Nitika Sharma, Gaurang P Nazar, Aastha Chugh, Mansi Chopra, Noreen D Mdege, Rijo M John, Monika Arora, Anup Karan

**Affiliations:** HRIDAY, New Delhi, India; HRIDAY, New Delhi, India; HRIDAY, New Delhi, India; HRIDAY, New Delhi, India; Department of Health Sciences, University of York, York, UK; Centre for Research in Health and Development, York, UK; Rajagiri College of Social Sciences, Kochi, Kerala, India; HRIDAY, New Delhi, India; Public Health Foundation of India, Gurugram, India; Public Health Foundation of India, Gurugram, India; Indian Institute of Public Health, Delhi, India

## Abstract

**Introduction:**

We studied the change in affordability of tobacco products, an important determinant of tobacco use, across the different socio-economic status (SES) in India.

**Aims and Methods:**

We calculated affordability in the form of relative income price (RIP-cost of tobacco products relative to income) for the years 2011–2012 and 2018–2019 using three different denominators, that is per capita gross domestic product (GDP) and net state domestic product at national and state levels, respectively; monthly per capita consumer expenditure (MPCE); and individual wages. We investigated RIP for cigarettes, bidis, and smokeless tobacco (SLT) across different SES groups (caste groups, type of employment, and education).

**Results:**

RIP increased marginally for cigarettes, bidis and remained almost constant for SLT across casual workers. However, when RIP was adjusted with SES variables, there was no significant change (*p* > .05) in the affordability of products for casual workers in the year 2018–2019 as compared to 2011–2012. For regular workers, cigarettes and bidis became marginally less affordable (*β* < 1), whereas affordability remained constant for SLT. All products became more affordable for backward caste groups within regular workers. When RIP was calculated using MPCE all tobacco products became less affordable in the year 2018–2019. However, after adjusting for SES variables SLT reported no change in affordability. There was a marginal increase in affordability for all products when RIP was calculated with GDP.

**Conclusions:**

Although implementation of GST has increased the price of tobacco products, it is still not sufficient to reduce the affordability of tobacco products, particularly SLT and especially for the lower SES group.

**Implications:**

Tobacco use and economic disadvantage conditions of the population are intricately linked. Affordability of tobacco products is influenced by socio-economic indicators like age, sex, income, education, etc. The literature measuring the affordability of tobacco products across different SES groups is scant in India. Additionally, existing literature measures affordability of tobacco products based on per capita GDP as a proxy for income. This is the first study in Indian context to report the change in affordability of tobacco products across different SES groups after adjusting for SES indicators, using individual-level income data. We have calculated the change in affordability of tobacco products between the year 2011–2012 and 2018–2019 using GDP, household income, and individual wages as a proxy for income.

## Introduction

The majority of the world’s tobacco users reside in low- and middle-income countries (LMICs).^1^ The economic burden from tobacco use is enormous and more catastrophic in LMICs than in high-income countries.^2^ Imposing high taxes on tobacco products (ie up to 75% of the retail price) is one of the most cost-effective measures to prevent tobacco use, especially among the young and the poor.^1,3^ Nevertheless, the impact of taxation on the price of tobacco products can be reduced if the income of consumers increases significantly.^4^ Hence, recent literature suggests that the concept of affordability of tobacco products is more relevant as compared to the real price of tobacco products when evaluating fiscal tobacco control policies.^5,6^ The affordability of tobacco products, is defined as the price of tobacco products relative to the income of consumers (relative income price [RIP]).^7^ The affordability index/RIP of tobacco products can be calculated using gross domestic product (GDP) per capita,^7^ household expenditure,^8^ or individual level income^9^ as a proxy for consumer income.^10^ The increase in price of tobacco products should outpace the increase in income of consumers for effective tobacco control.^10,11^ The per capita GDP data represents the overall aggregate economic activity data of the country, and often leads to biased affordability index results. The individual income data represents the closer measure of income while calculating the affordability of products but the information from national economic surveys is often limited to the employed sector.^5,9^

While there is an inverse relation between affordability index/RIP and consumption of tobacco products,^10,12^ affordability of tobacco products is also influenced by socio-economic indicators such as age, sex, income, education, etc.^10,13^ It is thus essential to understand the affordability of tobacco products across different socio-economic groups in the LMICs, especially in South–East Asian countries such as India where there is rapid economic growth and wide socio-economic disparities.^14–17^ Tobacco use and economic disadvantage conditions of population are intricately linked.^18,19^ In addition, in countries such as India the availability of a wide variety of tobacco products and wide disparity across their prices, impose major challenges in implementing fiscal tobacco control strategies.^20,21^

Before the implementation of the Goods and Services Tax (GST) in 2017, India’s central government levied an excise tax on tobacco products based on their characteristics (eg stick length and presence/absence of a filter), whilst state governments levied value-added tax (VAT). However, in July 2017 all these multiple taxes were subsumed under the Goods and Service Tax (GST),^22^ where all tobacco products were taxed at a fixed rate of 28%. The GST also includes an additional GST *compensation cess* (tax levied to compensate the state for loss of revenue in implementing GST as the GST is a consumption-based tax)^23^ on cigarettes and smokeless tobacco.

Although there is literature measuring the affordability of tobacco products across different data periods at the national and sub-national levels in India,^8,24–26^ the literature measuring the affordability of tobacco products across different SES groups is scant. Also, existing literature only measures affordability based on per capita GDP as a proxy for income.^8,24–26^ To the best of our knowledge, this is the first study in India to investigate the affordability of tobacco products using individual incomes, especially after the implementation of GST. We also investigated the change in affordability of tobacco products, adjusting for other SES indicators (age, sex, education, household type, and religion), across the different population and occupational groups (caste groups and type of employment) in India.

## Methods

### Data Sources

For the purpose of this study, we collected secondary data from several sources. The price data of tobacco products in India were collected from Labour Bureau, Ministry of Labour and Employment (MoLE), and the Government of India. Labour Bureau publishes monthly data on retail prices of different commodities, including smoking and smokeless tobacco products.^27^ Labour Bureau collects monthly price data from approximately 70 industrial centers spread across several states in India (24 states) for the purpose of constructing the Consumer Price Index for Industrial Workers (CPI-IW).

The National Sample Survey Office (NSSO), Ministry of Statistics and Programme Implementation (MoSPI), Government of India conducted a quinquennial survey named the Employment and Unemployment Survey (EUS) that provides information on the daily/monthly wage of the population employed for wage earnings in India, until 2011–2012.^28^ The Periodic Labour Force Survey (PLFS) conducted by the NSSO each year since 2017–2018, provides similar information on wages and earnings.^29^ Data from these two surveys (ie EUS and PLFS) in 2011–2012 and 2018–2019, respectively were used in the current study to estimate the per capita income and individual wages of the population as they are comparable in terms of sample design, thematic areas covered, and approaches to data collection. These surveys use a stratified multistage design and collect data from all the Indian states and Union Territories. The sample size of EUS 2011–2012 was 101 724 households (59 700 rural and 42 024 urban). Similarly, PLFS 2017–2018 collected data from 101 579 households (55 812 rural and 45 767 urban). In addition to labor market-related information, both surveys provided information on a range of socio-economic indicators of population. For instance, both surveys collected data on the total consumption expenditure of households, which we used to estimate “monthly per person consumption expenditure” (MPCE) as a measure for household income. We also used MPCE data to classify households in four equal (quartiles) groups. In addition, we used information on caste (social stratification), the major source of livelihood, and education of the head of households, in our affordability analysis for different socio-economic groups.

### Measures and Data Analysis

#### Price of Tobacco Products

The price of different brands and pack of tobacco products (for both the data periods 2011–2012 and 2018–2019) were first converted into their unit price and then price of 100 units of product (100 sticks each for cigarettes and bidis; and 100 grams of smokeless tobacco) was calculated. In case of multiple brands of the same product from the same state, we calculated average retail price of the product across different brands. Due to the heterogeneity of smokeless tobacco data, we only included chewing tobacco, and *zarda, kimam, surti* in the smokeless category in the present analysis.

#### Wages and Earnings

Data on wages and earnings of workers are available in EUS and PLFS on weekly basis for casual wage earners and monthly basis for workers employed on a regular basis. The survey sets also provides the actual number of working days for workers in a week. We multiplied the weekly earnings of workers by 4.3 (assuming a month has 4.3 weeks on average) to calculate the monthly wages for casual workers. The method, hence, only considers actual wages/earnings of workers based on actual days of working during the week in reference and hence during the month.

#### Monthly Per Capita Consumer Expenditure

The household total consumption expenditure was divided by the total number of household members to obtain per capita consumer expenditure. We used monthly per capita consumer expenditure (MPCE) as a proxy for per capita household income.

#### Per Capita GDP and Net State Domestic Product

The data on per capita GDP and net state domestic product (NSDP) were collected from the Reserve Bank of India.^30^

#### Relative Income Price

RIP defines tobacco product affordability as the percentage of income required to buy 100 packs of cigarettes or unit of tobacco. The higher the RIP, the less affordable are tobacco products, and vice versa. We calculated the RIP as the percentage of consumer income required to purchase a pack of 100 units of tobacco product (100 sticks for bidis and cigarettes; 100 grams of SLT product). This was calculated separately for years 2011–2012 and year 2018–2019, using three different methodologies from Blecher et al., (per capita GDP)^7^ Guindon et al., (household income)^8^ and Kan et al., (individual wages)^9^ respectively, both at the national and sub-national levels. The GDP/NSDP data was available annually during the period. However, MPCE and wages data are available from the surveys for 2011–2012 and 2018–2019. We used wages and MPCE data mainly because these two indicators were available for socio-economic groups, while GDP/NSDP data were not available for socio-economic groups. The additional definition and details of the variables are provided in the supplementary file (Supplementary Table S1).

Since the present analysis used survey data, we used survey weights in calculating the affordability index and in investigating the change in RIP across the different SES groups. We first calculated RIP only using price of tobacco products and income indicators. However, the changes in RIP may be confounded by a range of socio-economic status (SES) factors of households and tobacco consumers, we also estimated the affordability index at the national level adjusting for SES. Caste represents the social stratification system in India,^31^ and therein influences the economic activity as well access to public resources in India.^32^ Therefore, socio-economic factors we considered are caste status of households (4 groups: Scheduled Castes [SCs], Scheduled Tribes [STs], Other Backward Classes [OBCs], and Other [comparison group/ general caste groups]); and quintile groups of households (4 equal groups of 25% each) based on MPCE. STs are the tribal communities/tribes, SCs are the socially and economically deprived castes in the past and OBCs are the socially and economically marginalized castes that do not fall under the ambient of STs or SCs. We also used education as a SES group. The education was divided into three categories: illiterate, those with primary to higher secondary education and diploma/graduates/postgraduates (Supplementary Table S1). We used pooled linear regression model with RIP as the dependent variable and a range of SES indicators (age, sex, religion, and household type.) as independent variables. For estimating the changes in the RIP, we pooled the 2011–2012 and 2018–2019 data together and estimated time-interacted SES indicators while controlling for other SES and state-level fixed effects. However, due to extreme multi-collinearity, we did not include the education of the head of household (customary head of the household or the person in formal charge of the management of the household) as a time-interacted SES variable but rather adjusted it as a SES indicator in calculating RIP with MPCE. The regression specification^33,34^ for the adjusted estimates is presented in Equation 1


$$Y_{ijt}=\alpha +dt+\sum_{q=1}^{4}Q_{q}\beta_{1}q+\sum_{q=1}^{4}t*Q_{q}\beta_{2q}+\sum_{c=1}^{4}Caste_{c}\beta_{3c}+\sum_{c=1}^{4}t*Caste_{c}\beta_{4c}\;+\sum_{e=1}^{3}Edu_{e}\beta_{4c}\sum_{e=1}^{3}t*Edu_{e}\beta_{4c}+X_{ijt}\gamma +\eta_{j}+\xi it$$ (1)

In Equation (1) “y*_ijt_*” is the affordability index of a group of population “i” living in state “j” in time period “t” affiliated to a particular socio-economic characteristic (q for four quartile groups and c for four caste groups and e for three educational groups). Time dummy (2018–2019 = 1; 2011–2012 = 0) is indicated by “*dt*”. The predictor of affordability index, wealth quartile, caste, and education are represented by indicators Q, Caste, and Education respectively. The magnitude and the direction of changes in the affordability index by the socio-economic gradients during the survey periods was determined by the coefficients of the interaction terms between the survey periods and the predictors. X_ijt_ represents vector of other SES such as main source of livelihood (in four categories: self-employed, regular wage/salary earning, casual labor, others), education of head of households (in three categories), religion (two categories), sex (two categories), age (three categories), sector (two categories), adult proportion (continuous). State level fixed effect error term is represented by “$\eta_{j}$” and “ξit” represent usual stochastic error term. Standard errors were clustered at village level. We also conducted separate subgroup analysis for casual wage earners and regular wage earners to take care of occupational categories. However, in the occupational group analysis we didn’t consider quintile groups as a predictor as overwhelming majority of the individuals in the casual wage group belonged to the poorest quartile.

## Results

Table 1 summarizes the prices of tobacco products, GDP per capita, per capita MPCE, and individual wages for years 2011–2012 and 2018–2019. The price of 100 sticks of cigarettes was more than twice in the year 2018–2019 (584 INR) as compared to the year 2011–2012 (271.6 INR). Similarly, the price of bidi was slightly more than twice in the year 2018–2019 as compared to the year 2011–2012 (67.6 INR vs. 32.9 in 2011–2012). The price of SLT was 170.6 INR in the year 2018–2019 as compared to 95.9 INR in the year 2011–2012. MPCE increased by 35% (at current prices) in the year 2018–2019 as compared to year 2011–2012. There was a slightly less than double increase (at current prices) in the wages of casual workers in the year 2018–2019 (6211 INR/month) when compared to the year 2011–2012 (3492 INR). The wages for regular workers also increased from 10 951 INR/month in year 2011–2012 to 16 127 INR/month in year 2018–2019.

**Table 1.** Overview of the Price of Tobacco Products and Income of Consumers Across the Year 2011–2012 and 2018–2019

Variable	2011–2012	2018–2019
MPCE (INR)	1749	2367
GDP per capita (INR)	65 927	134 504
Regular worker wage per month(INR)	10 951	16 127
Casual worker wage per month (INR, estimates based on actual number of working days in a month)	3492	6211
Casual worker wage per day (INR)	141	277
Average price of 100 sticks of Cigarette (INR)	271.56	584
Average price of 100 sticks of Bidi (INR)	32.86	67.59
Average price of 100 grams of SLT (INR)	95.88	170.58

Data collected from E&U 2011–2012, PLFS 2018–2019; RBI 2011–2012, and 2018–2019, Labor Bureau 2011–2012, 2018–2019.

Table 2 presents the RIP for all the tobacco products in year 2011–2012 and 2018–2019, with all three denominators at the national level. The results when RIP was calculated using individual (monthly) earning measures as a denominator, indicate that RIP of cigarettes increased for regular workers (3.71% vs. 2.51%) as well casual workers (9.43% vs. 7.77%) in year 2018–2019 as compared to year 2011–2012. Similarly, RIP for bidis marginally increased for casual workers (1.11% vs. 0.96%) as well as for regular workers (0.45% vs. 0.32%) in 2018–2019. However, the RIP for smokeless tobacco products almost remained constant for casual workers in 2018–2019 compared to 2011–2012 (2.78% vs. 2.76%) but increased for the regular worker (1.11% in year 2018–2019 vs. 0.88% in year 2011–2012). There was a marginal increase in RIP for all the products when RIP was calculated with NSDP (Supplementary Table S2). Whereas all the tobacco products had become less affordable in year 2018–2019 as compared to 2011–2012 when the RIP was calculated using MPCE. The decline in affordability was most prominent when RIP was calculated using MPCE as a denominator.

**Table 2.** RIP (%) of Tobacco Products at National Level, Using all the Three Measures of Income as a Denominator

Denominators	Cigarettes (100 sticks)		Bidis (100 sticks)		Smokeless tobacco (100 grams)	
	2011–2012	2018–2019	2011–2012	2018–2019	2011–2012	2018–2019
*Wages*						
-RRegular workers	2.51	3.71	0.32	0.45	0.88	1.11
-Casual workers	7.77	9.43	0.96	1.11	2.78	2.76
MPCE	15.53	24.72	1.88	2.86	5.48	7.79
GDP per capita	0.41	0.44	0.056	0.057	0.15	0.14

RIP = relative income price.

Table 3 presents the change in the affordability index of tobacco products between year 2011–2012 to year 2018–2019 at the national as well as subnational level, using wages as a denominator for RIP.

**Table 3.** Affordability Index (RIP) of Tobacco Products Based on Monthly Wages

RIP (%)	Cigarettes (100 sticks)						Bidis (100 sticks)						Smokeless Tobacco (CT, ZKS) (100 gms)					
	Casual			Regular			Casual			Regular			Casual			Regular		
	2011–2012	2018–2019	∆RIP	2011–2012	2018–2019	∆RIP	2011–2012	2018–2019	∆RIP	2011–2012	2018–2019	∆RIP	2011–2012	2018–2019	∆RIP	2011–2012	2017–2018	∆RIP
*India*	**7.77**	**9.43**	0.01*	**2.51**	**3.71**	1.2*	**0.96**	**1.11**	0.15*	**0.32**	**0.45**	+0.13*	**2.78**	**2.76**	−0.02*	**0.88**	**1.11**	+0.23*
Jammu and Kashmir	4.65	4.85	0.2	1.96	1.96	**0**	—	—	NA	—	—	NA	5.58	—	NA	2.35	—	NA
Himachal Pradesh	6.86	8.92	2.06*	2.61	3.65	1.04*	0.46	0.54	0.08*	0.18	0.22	+0.05*	0.82	—	NA	0.31	—	NA
Punjab	5.680	9.80	4.12*	3.18	4.84	1.66*	0.65	0.94	0.29*	0.36	0.47	+0.11*	5.05	6.67	1.62*	2.82	3.29	+0.53*
Chandigarh	5.21	8.75	3.54*	q.78	2.73	0.95*	0.53	1.01	0.48*	0.18	0.31	+0.13*	0.47	0.54	0.07	0.16	0.17	0.01
Haryana	3.22	8.73	5.51*	0.99	4.12	3.13*	0.31	0.65	0.34*	0.09	0.31	+0.22*	0.56	0.54	−0.02*	0.17	0.25	+0.08*
Delhi	4.29	5.96	1.67	1.74	3.19	1.45*	0.51	0.56	0.05	0.20	0.31	+0.1*	0.19	—	NA	0.08	—	NA
Rajasthan	7.36	9.74	2.38*	2.93	4.19	1.26*	1.07	1.12	0.05	0.42	0.48	+0.06*	4.92	3.85	−1.07*	1.96	1.66	−0.26*
Uttar Pradesh	8.47	9.04	0.57*	2.56	3.54	0.98*	0.72	0.91	0.19	0.22	0.36	+0.15*	2.98	4.21	1.23*	0.90	1.65	+0.77*
Bihar	7.03	7.07	0.04*	2.07	3.81	1.74*	0.57	0.43	−0.14*	0.17	0.23	+0.07*	0.67	0.57	−0.10*	0.19	0.31	+0.12*
Tripura	7.93	7.59	−0.34	2.91	2.52	−0.39*	0.84	1.02	0.18*	0.31	0.34	0.05	3.85	5.09	1.24*	1.41	1.69	+0.31*
Assam	5.75	4.96	−0.79*	1.97	2.99	1.02*	0.76	0.76	0	0.26	0.46	+0.2*	0.89	0.86	−**0.03**	0.31	0.52	+0.22*
West Bengal	9.03	9.50	0.47*	2.63	4.35	1.72*	0.76	0.84	0.08*	0.22	0.39	+0.17*	2.98	2.41	−0.57*	0.87	1.1	+0.25*
Jharkhand	7.09	10.45	3.36*	1.69	3.53	1.84*	0.58	0.82	0.24*	0.14	0.28	+0.15*	5.83	8.03	2.20*	1.39	2.71	+1.36*
Odisha	9.71	9.69	−0.02	2.84	3.14	0.3	0.98	1.04	0.06	0.29	0.34	+0.06*	0.90	0.83	−0.07*	0.26	0.27	0.01
Chhattisgarh	12.62	14.95	2.33*	3.42	4.32	0.9	1.78	1.82	0.04	0.48	0.52	0.05	2.72	0.98	−1.74*	0.74	0.28	−0.44*
Madhya Pradesh	10.18	13.76	3.58*	2.68	4.33	1.65	1.36	1.68	0.32*	0.36	0.53	+0.18*	0.71	0.44	−0.27*	0.19	0.14	−0.04*
Gujarat	9.80	11.99	2.19*	3.73	4.28	**0.55**	1.28	1.48	0.20*	0.49	0.53	+0.05*	2.56	1.71	−0.85*	0.97	0.61	−0.34*
Maharashtra	9.53	12.43	2.90*	2.23	3.59	1.36*	1.22	1.43	0.21*	0.29	0.41	+0.13*	2.41	3.64	1.23*	0.56	1.06	+0.51*
Andhra Pradesh	8.40	9.34	0.94*	3.29	3.37	0.08*	1.09	1.34	0.25*	0.43	0.49	+0.12*	7.87	5.99	−1.88*	3.09	2.16	−0.53*
Karnataka	6.85	8.14	1.29*	2.38	2.89	0.51*	1.09	1.22	0.13	0.38	0.43	+0.06*	2.54	1.74	−0.80*	0.88	0.62	−0.24*
Goa	6.24	6.84	0.6	22.58	3.49	0.91*	0.79	0.95	0.16	0.33	0.49	+0.17*	0.41	—	NA	0.17	—	NA
Kerala	4.26	5.57	1.31*	2.72	2.94	0.22*	0.67	1.28	0.61*	0.43	0.68	+0.26*	0.47	—	NA	0.30	—	NA
Tamil Nadu	6.96	10.32	3.36*	2.85	4.31	1.46	1.29	1.62	0.33*	0.53	0.68	+0.16*	0.51	—	NA	0.21	—	NA
Puducherry	2.94	9.88	6.94*	1.21	**3.46**	2.25*	1.03	1.38	0.35*	0.43	0.48	0.06	0.39	0.41	0.02	0.16	0.14	−0.02*

All the bold values have *p* values <.05.

Authors’ calculation.

−Reduction in affordability index, i.e. products have become relatively more affordable.

*p value less than .05.

CT: Chewing tobacco; ZKS: *Zarda, kimam, surti.*

∆RIP: Change in RIP (RIP 2018–2019 to RIP 2011–2012).

RIP = relative income price

At the sub-national level, cigarettes became relatively less affordable across eighteen states in 2018–2019 for casual workers including Punjab, Haryana, Himachal Pradesh, Chandigarh, Jharkhand, Chhattisgarh, Madhya Pradesh, Gujarat, Maharashtra, etc. (Table 3). Bidis became relatively less affordable for 14 states in 2018–2019 for casual workers. Whereas SLT became relatively less affordable for five states for casual workers in 2018–2019 as compared to year 2011–2012. However, when we used MPCE and NSDP in calculating RIP most of the states shows the decline in affordability, more so when we considered wages as a denominator (Supplementary Table S2–S3).

Using Equation 1, Table 4 presents the unadjusted as well as adjusted coefficients of changes in RIP of tobacco products when calculated using individual (monthly) wage measures. The adjusted RIP coefficients were calculated across different caste and education groups, after adjusting for other SES indicators like age, gender, main source of livelihood, religion, and states. The regression analysis results suggest that although RIP marginally increased for cigarettes and decreased for bidis and SLT, there was no significant change (*p* > .05) in the affordability of products for casual workers in year 2018–2019 as compared to year 2011–2012. For regular workers, cigarettes and bidis became marginally less affordable in 2018–2019 as compared to year 2011–2012 (*β* < 1). Furthermore, there was no change in affordability for SLT across regular workers.

**Table 4.** Estimated Coefficients of OLS Regression Analysis for RIP of Cigarettes, Bidis, and Smokeless Tobacco for the Given Data Points (2011–2012 and 2018–2019) using Monthly Wages

RIP (%)	Cigarettes		Bidis		Smokeless tobacco	
	Unadjusted coefficient (S.E)	Adjusted coefficient (S.E)	Unadjusted coefficient (S.E)	Adjusted coefficient (S.E)	Unadjusted coefficient (S.E)	Adjusted coefficient (S.E)
i.Casual workers						
*Time*						
Year 2011–2012	Reference	Reference	Reference	Reference	Reference	Reference
Year 2018–2019	1.371 (0.096)***	0.621(0.750)	0.130 (0.012)***	−0.099 (0.099)	−0.277 (0.051)***	−0.649 (0.338)
*Caste groups*						
Other caste groups	Reference	Reference	Reference	Reference	Reference	Reference
Schedule tribe*2018–2019	1.720 (0.364)***	1.432 (0.911)	−0.022 (0.047)	0.0472 (0.110)	−0.139 (0.188)	0.124 (0.292)
Schedule caste*2018–2019	1.324 (0.297)***	0.916 (0.515)	0.039 (0.039)	0.065 (0.054)	0.365 (0.157)**	0.286 (0.216)
Backward caste *2018–2019	1.994 (0.283)***	1.453 (0.520)***	0.112 (0.037)***	0.130 (0.060)**	0.925 (0.150)***	0.365 (0.238)
Education						
Diploma/graduate/postgraduate*2018–2019	Reference	**Reference**	**Reference**	Reference	**Reference**	Reference
Illiterate (including less than primary)*2018–2019	−0.394 (0.861)	0.328 (0.721)	−0.105 (0.111)	0.027 (0.098)	−1.222 (0.495)**	0.195 (0.296)
Primary to higher secondary*2018–2019	−1.265 (0.861)	−0.401 (0.703)	−0.174 (0.111)	−0.028 (0.094)	−0.843 (0.496)	−0.048 (0.268)
Constant	11.55 (0.158)***	6.938 (2.629)***	1.250 (0.0206)***	**0.305 (0.257)**	4.113 (0.081)***	7.664 (0.971)***
Observations	53 641	53 623	52 160	52 142	48 428	48 411
R^2^	0.010	0.142	0.010	0.214	0.004	0.334
ii.Regular workers						
*Time*						
Year 2011–2012	Reference	Reference	Reference	Reference	Reference	Reference
Year 2018–2019	0.170 (0.079)**	0.719 (0.223)***	−0.058 (0.010)***	0.078 (0.031)***	−0.239 (0.038)***	−0.163 (0.137)
*Caste groups*						
Other caste groups	Reference	Reference	Reference	Reference	Reference	Reference
Schedule tribe*2018–2019	−0.056 (0.387)	−0.104 (0.830)	−0.117 (0.051)**	−0.091 (0.106)	−0.122 (0.178)	−0.071 (0.291)
Schedule caste*2018–2019	0.09 (0.229)	−0.079 (0.504)	−0.064 (0.030)**	−0.044 (0.059)	−0.122 (0.178)	−0.570 (0.228)
Backward caste*2018–2019	−0.777 (0.179)***	−0.768 (0.351)**	−0.145 (0.023)***	-0.116 (0.045)**	0.001 (0.056)	−0.382 (0.182)**
Education						
Diploma/graduate/postgraduate*2018–2019	Reference	Reference	Reference	Reference	Reference	Reference
Illiterate (including less than primary)*2018–2019	0.255 (0.252)	0.523 (0.760)	−0.049 (0.033)	−0.008 (0.086)	−0.490 (0.122)***	−0.263 (0.325)
Primary to higher secondary*2018–2019	-0.948 (0.168)***	−0.818 (0.234)***	−0.189 (0.022)***	−0.152 (0.034)***	−0.381 (0.081)***	−0.327 (0.129)**
Constant	5.564 (0.091)***	3.070 (3.061)	0.659 (0.012)***	0.056 (0.333)	2.050 (0.041)***	5.606 (1.357)***
Observations	70 683	70 659	67 256	67 233	62 263	62 239
*R* ^2^	0.012	0.132	0.016	0.137	0.009	0.212

All the bold values have *p* values <.05.

***p values less than .001 and **p value less than .05.

− Adjusted coefficient (after adjusting all the SES indicators); unadjusted coefficient (without adjusting other SES indicators).

− The regression analysis controls for the following factors: Age (less than 15 years, 15–49 years, 50 and above), main source of livelihood, education, sex, religion, states; clustered at village level. For the comprehensive presentation of results the estimates for these variables are not presented in this table.

−The interaction variables (Caste*2018–2019 and Education*2018–2019) were created using a dummy variable and then incorporated in the analysis.

RIP = relative income price.

Cigarettes became significantly less affordable for OBCs as compared to other caste groups (*p* < .05) within casual workers. Contrastingly, all the tobacco products became more affordable for OBCs group as compared to other caste groups in 2018–2019, within regular workers (*p* < .05). Similarly, all products were more affordable across those with formal education (primary to higher secondary) within regular workers (Table 4).

The OLS regression models were also used to estimate the affordability index of tobacco products, with MPCE (details given in Supplementary Table S4). The results showed that while cigarettes and bidi became relatively less affordable, SLT reported no change in their affordability in 2018–2019 as compared to 2011–2012. Further, across all the wealth quartiles, all tobacco products became relatively less affordable. However, there was no change in the affordability of bidis and SLT across different caste groups. Cigarettes became less affordable across backward caste group (*p* < .05) (Supplementary Table S4).

## Discussion

The existing literature on affordability of tobacco products has either used household income^35^ or per capita GDP^24,25^ as a proxy for income in calculating affordability of tobacco products, in India. This is the first study in Indian context to report affordability index of tobacco products based on individual level income data, especially after the implementation of GST. The findings of the study suggest that there was an increase in the price of tobacco products, and RIP increased marginally for cigarettes, bidis, and remained almost constant for SLT across casual workers. However, there was no change in affordability of all tobacco products across casual workers when RIP was adjusted across different SES indicators. Similarly, there was no change in affordability of SLT across regular workers as well. Cigarettes and bidis became marginally less affordable (*β* < 1) for regular workers in 2018–2019, as compared to 2011–2012. Furthermore, all tobacco products became more affordable across OBCs and those with formal education within regular workers. When the RIP was calculated using MPCE as a proxy for income, the results suggested that products became relatively less affordable in 2018–2019. However, there was no significant change in bidis and SLT across different caste groups. Whereas there was a marginal increase in RIP across all tobacco products when calculated using GDP as a proxy for income. It can be argued that RIP when calculated with MPCE as a denominator, can provide an underestimate of income since tobacco products constitute a small proportion of household consumer expenditure. Additionally, due to the existing tobacco control activities, there is a chance that households do not fully report their tobacco expenditure. Previous literature suggests that average income as a denominator can often lead to overestimation of affordability index, especially in the LMICs,^5,9^ therefore, individual wages represent a closer proxy for actual income.

After the introduction of GST, all the VAT and excise duty are now included under 28% GST regime.^22^ Our findings align with those reported in a study by Goodchild et al., which found no change in affordability for bidis and cigarettes at the national level in India post-GST implementation.^24^ Another study by John et al., however, suggested that all tobacco products have become more affordable over a decade (from 2007–2008 to 2018–2019).^23^ Nevertheless, this evidence together suggests that any increases in the price of all tobacco products over the past decade, including after GST implementation, have not resulted in a significant decrease in affordability, especially across the lower SES group (casual workers, STs/SCs/BCs, and illiterate). This could be due to increase in individual wages which might have outweighed the increase in tobacco taxes. Another noteworthy point is that in India, under the Mahatma Gandhi National Rural Employment Guarantee Act (MNREGA), it is compulsory to provide 100 days (per year) wage to casual workers, which might have led to all tobacco products becoming more affordable for this population group (Supplementary Table S5). This income adjustment should have been made while deciding GST for sin products such as tobacco. Furthermore, the absence of revision in GST on cigarettes, and compensation cess on bidis is likely to only aggravate the increasing affordability of tobacco products.^25^

The Global Adult Tobacco Survey (GATS) conducted in 2016/17 in India found that the prevalence of tobacco use across products was higher in the lower SES compared as to high SES groups.^36^ The increase or no change in affordability of tobacco products for the lower SES group (eg casual workers and SCs/STs/OBCs in India) is particularly of concern from public health and sustainable development perspective for India and other LMICs, as these groups comprise a huge proportion of the population and are vulnerable to bear the burden of tobacco attributed morbidity and mortality.^37^ Additionally, the heterogeneity across types and prices of tobacco products (especially SLT) encourages product substitution and thereby undermining the efficacy of tobacco taxes.^3,6^ This suggests the need for the implementation of higher and uniform taxes across tobacco products to curb tobacco use and protect the vulnerable poor.

At the state level, a vast disparity in affordability was observed across cigarettes for casual workers. Earlier studies have also suggested the differential impact of GST at the subnational level.^8,24–26^ The variation in affordability can be attributed to the possible income disparity across the states. Besides that the transportation cost incurred on exporting the tobacco products from producer to consumer states can also contribute to the disparity of affordability of tobacco products at the subnational level.^26^

Taxation is a critical intervention for tobacco control and is closely linked to affordability. However, the tax structure in India is complex with multiple tax rates across different tobacco products. Applying specific rather than *ad valorem* tax rates and cess to low-price products especially *bidis* (whose consumption is increasing as per GATS India survey) may ensure an increase in prices and a decrease in affordability.^24^ To effectively reduce the affordability of tobacco products, and hence their use, the tobacco product price increases must outpace the income growth of the consumers. In addition, taxation must be accompanied by strong tax enforcement and administration; simultaneous regulation and comprehensive licensing system of the tobacco supply chain especially for low-priced tobacco products; and strict control of tobacco industry interference and tactics. The evidence obtained from this paper provides a strong basis for further increasing tobacco taxation in India, taking individual incomes into account and adopting a public health lens to protect the vulnerable poor.

### Strengths and Limitations

Although this is the first study to investigate the affordability index based on SES, for tobacco products in India using individual wages both for casual and regular workers, the study has certain limitations. The penetration of CPI-IW data might be limited to certain rural or urban areas of the country and hence, might not be a true representative of the average price of tobacco products. The data does not represent the average price for cigarettes across different price tiers. Additionally, due to the large heterogeneity of SLT products within the country we used chewing tobacco and *zarda, kimam, surti* for analysis, since these products are consumed in the majority of the states. We also used individual wages as income in calculating affordability index which might only represent affordability of products among the working population and not among those who are unemployed or self-employed. However, the objective of our study was to study the change in affordability of tobacco products across the different SES groups and hence, we do not expect these limitations to create any major bias in our results.

## Conclusion

Our study suggests that although the implementation of GST has increased the price of tobacco products, these increases have not been effective in sufficiently reducing the affordability of tobacco products, especially for the lower SES group. To be effective, tax increases should outpace income growth. Furthermore, there is a need for increased and uniform taxation across all tobacco products for successful and sustainable tobacco control in India and other LMICs, since this is the most cost-effective tobacco control policy intervention to curb tobacco use among vulnerable populations in LMICs.

## Supplementary Material

A Contributorship Form detailing each author’s specific involvement with this content, as well as any supplementary data, are available online at https://academic.oup.com/ntr.

ntac230_suppl_Supplementary_File

ntac230_suppl_Supplementary_Taxonomy-form

## Data Availability

All the concerned data is available in the manuscript and its online supplementary material. All the datasets were derived from sources present in the public domain: http://labourbureaucpi.gov.in; http://microdata.gov.in/nada43/index.php/catalog/EUE; https://mospi.gov.in/web/plfs; https://www.rbi.org.in.
