# Supplementary material for: Socio-Economic Status Differences in Changing Affordability of Tobacco Products from 2011–2012 to 2018–2019 in India
Source: Nicotine Tob Res. 2022 Oct 4;25(4):709–17. doi: 10.1093/ntr/ntac230 (PMC10032189; doi:10.1093/ntr/ntac230)
Supplement: ntac230_suppl_Supplementary_File [file ntac230_suppl_supplementary_file.docx]

**Table S1: Variables used in the analysis**

| **Affordability index (RIP%)**  $\frac{Price of tobacco products (100 units)}{Income}$* 100 | % age of consumer income required to purchase 100 units of tobacco products. The higher the RIP, the less affordable are tobacco products, and vice versa |
| --- | --- |
| ***Regular worker***:  ***Casual worker:*** | These were persons who worked in others‟ farm or nonfarm enterprises (both household and non-household) and, in return, received salary or wages on a regular basis (i.e. not on the basis of daily or periodic renewal of work contract). This category included not only persons getting time wage but also persons receiving piece wage or salary and paid apprentices, both full time and part-time.  A person who was casually engaged in others‟ farm or non-farm enterprises (both household and non-household) and, in return, received wages according to the terms of the daily or periodic work contract, was considered as a casual workers. These also include workers those under public work , i.e, those activities which were sponsored by Government or Local Bodies, and which cover local area development works like construction of roads, dams, bunds, digging of ponds, etc., as relief measures, or as an outcome of employment generation schemes under the poverty alleviation programmes such as Mahatma Gandhi National Rural Employment Guarantee (MGNREG) works, Sampoorna Grameen Rozgar Yojana  (SGRY), National Food for Work Programme (NFFWP), etc. |
| **Caste**  **Wealth quintiles** | The PLFS & E&U contains 4 categories for caste, schedule tribe, schedule caste, backward class and other caste groups.  The wealth quintiles are created based on the per capita monthly household consumer expenditure (proxy for income). Four wealth quintiles were generated: Poorest, second poorest and middle |
| Age (years) | Categorized in three categories:   - Below 15 years (reference) - 15-49 - 50 and above |
| Religion | The PLFS and E &U contains 8 categories for religion, however for present analysis we categorized it into Hindus and others ( Islam , Christianity , Sikhism, Jainism , Buddhism, Zoroastrianism , any other) |
| House hold type (Main source of livelihood) | The household type was further categorized into self-employed , regular wage/salary earning , casual labour , others. The household type was decided based on the sources of the household's income during the 365 days preceding the date of survey. For this purpose, only the household's  income (net income and not gross income) from economic activities was considered; but the incomes of servants and paying guests were not taken into account |
| Sex | Sex was categorized into males and females |
| Sector | Sector was categorized in urban and rural |
| Adult proportion | The proportion of adults present in the household |
| Education | Incase of income derived from house hold income it was categorized whether head of family is educated or illiterate. The various education categories in schedule were as follows: not literate -01, literate without formal schooling: EGS/ NFEC/ AEC -02, TLC -03, others -04; literate: below  primary -05, primary -06, middle -07, secondary -08, higher secondary -10, diploma/certificate course -11, graduate -  12, postgraduate and above -13. We categorized 1 to 5 under code ‘0’ (illiterate category); 6-10 under (education from primary to higher secondary coded as ‘1’; 11-13 under code ‘2’ for better fit of our models. |

**Table S2: Affordability index (RIP %) of tobacco products when calculated with GDP/NSDP as a proxy for income**

|  | **Cigarettes** | |  | **Bidis** | |  | **Smokeless tobacco** | |  |
| --- | --- | --- | --- | --- | --- | --- | --- | --- | --- |
|  | **2011-12** | **2018-19** | **Change in RIP** | **2011-12** | **2017-18** | **Change in RIP** | **2011-12** | **2018-19** | **Change in RIP** |
| **India** | **0.41** | **0.44** | **0.03*** | **0.056** | **0.057** | **0.001*** | **0.15** | **0.14** | -0.01 |
| Jammu & Kashmir | 0.47 | 0.44 | -0.03 | ---- | ----- | **NA** | 0.56 | ---- | NA |
| Himachal Pradesh | 0.35 | 0.38 | 0.03 | 0.03 | 0.02 | -0.01 | 0.04 | ---- | NA |
| Punjab | 0.34 | 0.45 | 0.11 | 0.04 | 0.04 | 0 | 0.30 | 0.31 | 0.01 |
| Chandigarh | 0.19 | 0.23 | 0.04 | 0.02 | 0.02 | 0 | 0.02 | 0.01 | -0.01 |
| Haryana | 0.17 | 0.34 | 0.17 | 0.02 | 0.03 | 0.01 | 0.03 | 0.02 | -0.01 |
| Delhi | 0.16 | 0.20 | 0.04 | 0.02 | 0.02 | 0 | 0.01 | ----- | NA |
| Rajasthan | 0.54 | 0.63 | 0.09 | 0.08 | 0.07 | -0.01 | 0.36 | 0.25 | -0.11 |
| Uttar Pradesh | 0.87 | 0.84 | -0.03 | 0.07 | 0.08 | 0.01 | 0.30 | 0.39 | 0.09 |
| Bihar | 1.14 | 1.42 | 0.28 | 0.09 | 0.09 | 0 | 0.11 | 0.12 | 0.01 |
| Tripura | 0.58 | 0.43 | -0.15 | 0.06 | 0.08 | 0.02 | 0.28 | 0.29 | 0.01 |
| Assam | 0.54 | 0.49 | -0.05 | 0.07 | 0.06 | -0.01 | 0.08 | 0.08 | 0 |
| West Bengal | 0.50 | 0.48 | -0.02 | 0.04 | 0.07 | 0.03 | 0.17 | 0.12 | -0.05 |
| Jharkhand | 0.63 | 0.77 | 0.14 | 0.05 | 0.04 | -0.01 | 0.51 | 0.59 | 0.08 |
| Odisha | 0.56 | 0.48 | -0.08 | 0.06 | 0.06 | 0 | 0.05 | 0.04 | -0.01 |
| Chhattisgarh | 0.52 | 0.66 | 0.14 | 0.07 | 0.05 | -0.02 | 0.11 | 0.04 | -0.07 |
| Madhya Pradesh | 0.73 | 0.71 | -0.02 | 0.09 | 0.08 | -0.01 | 0.05 | 0.02 | -0.03 |
| Gujarat | 0.33 | 0.33 | 0 | 0.04 | 0.09 | 0.05 | 0.09 | 0.05 | -0.04 |
| Maharashtra | 0.29 | 0.35 | 0.06 | 0.04 | 0.04 | 0 | 0.07 | 0.10 | 0.03 |
| Andhra Pradesh | 0.41 | 0.36 | -0.05 | 0.05 | 0.05 | 0 | 0.39 | 0.23 | -0.16 |
| Karnataka | 0.27 | 0.26 | -0.01 | 0.04 | 0.04 | 0 | 0.10 | 0.06 | -0.04 |
| Goa | 0.12 | 0.16 | 0.04 | 0.01 | 0.02 | 0.01 | 0.01 | Na | NA |
| Kerala | 0.28 | 0.26 | -0.02 | 0.04 | 0.06 | 0.02 | 0.03 | NA | NA |
| Tamil Nadu | 0.28 | 0.33 | 0.05 | 0.05 | 0.05 | 0 | 0.02 | NA | NA |
| Puducherry | 0.10 | 0.29 | 0.19 | 0.03 | 0.04 | 0.01 | 0.01 | 0.01 | 0 |

*** p-value less than 0.05**

**Table S 3: Affordability index (RIP %) of tobacco products when calculated with per capita household income as a proxy for income**

|  | **Cigarettes (100 sticks)** | | | | **Bidis (100 sticks)** | | | | **Smokeless tobacco (100 gms)** | | | |
| --- | --- | --- | --- | --- | --- | --- | --- | --- | --- | --- | --- | --- |
|  | **2011-12** | **2018-19** | **Change in RIP** | **P-value** | **2011-12** | **2017-18** | **Change in RIP** | **p-value** | **2011-12** | **2018-19** | **Change in RIP** | **p-value** |
|  |  |  |  |  |  |  |  |  |  |  |  |  |
| **India** | **15.53** | **24.72** | 9.19 | **0.00*** | **1.88** | **2.86** | 0.98 | **0.00*** | **5.48** | **7.79** | 2.31 | **0.00*** |
| Jammu & Kashmir | 14.25 | 16.20 | 1.95 | **0.00*** | --- | ---- | NA | **NA** | 17.10 | NA | NA | NA |
| Himachal Pradesh | 14.96 | 25.87 | 10.91 | **0.00*** | 1.01 | 1.55 | 0.54 | **0.00*** | 1.78 | NA | NA | NA |
| Punjab | 12.18 | 19.49 | 7.31 | **0.00*** | 1.39 | 1.88 | 0.49 | **0.00*** | 10.81 | 13.25 | 2.44 | 0.00* |
| Chandigarh | 9.17 | 11.65 | 2.48 | **0.00*** | 0.94 | 1.34 | 0.4 | **0.00*** | 0.83 | 0.72 | -0.11 | 0.08 |
| Haryana | 7.02 | 24.68 | 17.66 | **0.00*** | 0.68 | 1.84 | 1.16 | **0.00*** | 1.21 | 1.52 | 0.31 | 0.00* |
| Delhi | 8.28 | 14.94 | 6.66 | **0.00*** | 0.97 | 1.41 | 0.44 | **0.00*** | 0.38 | NA | NA | NA |
| Rajasthan | 18.15 | 30.57 | 12.42 | **0.00*** | 2.65 | 3.51 | 0.86 | **0.00*** | 12.15 | 12.09 | -0.06 | 0.88 |
| Uttar Pradesh | 21.07 | 29.72 | 8.65 | **0.00*** | 1.80 | 2.98 | 1.18 | **0.00*** | 7.41 | 13.83 | 6.42 | 0.00* |
| Bihar | 23.19 | 39.77 | 16.58 | **0.00*** | 1.88 | 2.39 | 0.51 | **0.00*** | 2.22 | 3.22 | 1 | 0.00* |
| Tripura | 19.12 | 19.37 | 0.25 | **0.61** | 2.01 | 2.60 | 0.59 | **0.00*** | 9.29 | 13.00 | 3.71 | 0.00* |
| Assam | 18.29 | 19.35 | 1.06 | **0.01** | 2.43 | 2.95 | 0.52 | **0.00*** | 2.85 | 3.35 | 0.5 | 0.00* |
| West Bengal | 16.35 | 22.08 | 5.73 | **0.00*** | 1.38 | 1.96 | 0.58 | **0.00*** | 5.39 | 5.60 | 0.21 | 0.19 |
| Jharkhand | 21.69 | 33.76 | 12.07 | **0.00*** | 1.77 | 2.66 | 0.89 | **0.00*** | 17.83 | 25.94 | 8.11 | 0.00* |
| Odisha | 24.81 | 28.58 | 3.77 | **0.00*** | 2.52 | 3.06 | 0.54 | **0.00*** | 2.30 | 2.46 | 0.16 | 0.01 |
| Chhattisgarh | 24.56 | 43.51 | 18.95 | **0.00*** | 3.47 | 5.29 | 1.82 | **0.00*** | 5.29 | 2.84 | -2.45 | 0.00* |
| Madhya Pradesh | 21.03 | 35.99 | 14.96 | **0.00*** | 2.81 | 4.39 | 1.58 | **0.00*** | 1.47 | 1.15 | -0.32 | 0.00* |
| Gujarat | 14.78 | 24.05 | 9.27 | **0.00*** | 1.94 | 2.96 | 1.02 | **0.00*** | 3.86 | 3.44 | -0.42 | 0.00* |
| Maharashtra | 12.69 | 24.77 | 12.08 | **0.00*** | 1.64 | 2.84 | 1.2 | **0.00*** | 3.21 | 7.26 | 4.05 | 0.00* |
| Andhra Pradesh | 14.84 | 19.12 | 4.28 | **0.00*** | 1.93 | 2.75 | 0.82 | **0.00*** | 13.91 | 12.27 | -1.64 | 0.00* |
| Karnataka | 11.77 | 20.38 | 8.61 | **0.00*** | 1.89 | 3.03 | 1.14 | **0.00*** | 4.37 | 4.36 | -0.01 | 0.96 |
| Goa | 11.49 | 16.82 | 5.33 | **0.00*** | 1.46 | 2.34 | 0.88 | **0.00*** | 0.76 | NA | NA | NA |
| Kerala | 10.27 | 15.48 | 5.21 | **0.00*** | 1.62 | 3.57 | 1.95 | **0.00*** | 1.13 | NA | NA | NA |
| Tamil Nadu | 12.90 | 20.33 | 7.43 | **0.00*** | 2.39 | 3.19 | 0.8 | **0.00*** | 0.93 | NA | NA | NA |
| Puducherry | 3.98 | 15.24 | 11.26 | **0.00*** | 1.39 | 2.12 | 0.73 | **0.00*** | 0.53 | 0.64 | 0.11 | 0.00* |

**Table S 4 :** **Estimated coefficients of OLS regression analysis for RIP of cigarettes, bidis and smokeless tobacco for the given data points (2011-12 and 2018-19) using MPCE**

|  | **Cigarettes** | | **Bidis** | | **Smokeless tobacco** | |
| --- | --- | --- | --- | --- | --- | --- |
|  | **Unadjusted coefficient (S.E)** | **Adjusted coefficient (S.E)** | **Unadjusted coefficient (S.E)** | **Adjusted coefficient (S.E)** | **Unadjusted coefficient (S.E)** | **Adjusted coefficient (S.E)** |
| **Time** |  |  |  |  |  |  |
| Year 2011-12 | Reference | Reference | Reference | Reference | Reference | Reference |
| Year 2018-19 | 13.05(0.087)*** | 5.746(0.655)*** | 1.323(0.0104)*** | 0.662(0.0704)*** | 2.965(0.046)*** | 0.674(0.756) |
| **Caste groups** |  |  |  |  |  |  |
| Other caste groups | Reference | Reference | Reference | Reference | Reference | Reference |
| Schedule tribe *2018-19 | -2.816(0.201)*** | 0.855(1.253) | 0.454(0.039)*** | -0.004(0.143) | 0.232(0.174) | -0.296(1.412) |
| Schedule caste*2018-19 | 4.00(0.315)*** | 1.051(0.605) | 0.209(0.029)*** | 0.095(0.077) | 1.291(0.133)*** | 0.938(0.725) |
| Backward caste *2018-19 | 1.200(0.229)*** | 1.744(0.583)*** | 0.238(0.024)*** | 0.144(0.077) | 1.867(0.110)*** | 1.564(0.881) |
| **Wealth quintiles** |  |  |  |  |  |  |
| Richest *2018-19 | Reference | Reference | Reference | Reference | Reference | Reference |
| Poorest*2018-19 | 29.32(0.167)*** | 36.31(2.157)*** | 3.209(0.021)*** | 3.965(0.353)*** | 7.425(0.130)*** | 8.186(2.761)*** |
| Second poorest*2018-19 | 15.93(0.149)*** | 19.36(1.109)*** | 1.597(0.019)*** | 2.018(0.168)*** | 3.754(0.116)*** | 4.969(1.685)*** |
| Middle *2018-19 | 8.250(0.146)*** | 10.18(0.785)*** | 0.921(0.018)*** | 1.143(0.092)*** | 2.058(0.113)*** | 2.646(1.029)*** |
| **Constant** | \| 0.946(0.081)* \| \| --- \| | \| 15.24(1.636)*** \| \| --- \| | \| 0.202(0.013) \| \| --- \| | \| 1.250(0.177)*** \| \| --- \| | \| 6.444(0.057)*** \| \| --- \| | \| 7.199(1.624)*** \| \| --- \| |
| **Observations** | 177200 | 46,990 | 170472 | 45484 | 159156 | 43200 |
| **R^2^** | 0.68 | 0.68 | 0.17 | 0.65 | 0.05 | 0.24 |

*****p value less than 0.01 ** p value less than 0.05**

The regression analysis controls for SES indicators like house hold type, sector, adult proportion, states; clustered at village level. For the comprehensive presentation of results the estimates for these variables are not presented in this table.

-The interaction variables (Caste *2018-19 and Wealth quintile *2018-19) were created using a dummy variable and then incorporated in the analysis.

**Table S5: Estimated coefficients of OLS regression analysis for affordability index of cigarettes, bidis and smokeless tobacco for the given data points (2011-12 and 2018-19) assuming that casual workers work for all the days in a month (using individual wage)**

| **RIP (%)** | **CIGARETTES** | | **BIDIS** | | **SMOKELESS TOBACCO** | |
| --- | --- | --- | --- | --- | --- | --- |
|  | **Unadjusted coefficient (S.E)** | **Adjusted coefficient (S.E)** | **Unadjusted coefficient (S.E)** | **Adjusted coefficient (S.E)** | **Unadjusted coefficient (S.E)** | **Adjusted coefficient (S.E)** |
| **Casual workers** | | | | | | |
| **Time** |  |  |  |  |  |  |
| Year 2011-12 | Reference | Reference | Reference | Reference | Reference | Reference |
| Year 2018-19 | **0.0929(0.0426)**** | **0.121(0.395)** | **-0.033(0.005)***** | **0.00003(0.050)** | **-0.431(0.026)***** | **-0.526(0.206)***** |
| **Caste groups** |  |  |  |  |  |  |
| Other caste groups | Reference | Reference | Reference | Reference | Reference | Reference |
| Schedule tribe *2018-19 | 0.106(0.161) | 0.017(0.435) | **-0.128(0.020)***** | **-0.074 (0.050)** | **-0.478(0.094)***** | -0.275(0.161) |
| Schedule caste*2018-19 | 0.587(0.132)*** | 0.375(0.265) | -0.013(0.017) | 0.009(0.026) | 0.122(0.0783) | 0.056(0.110) |
| Backward caste *2018-19 | 0.688(0.125)*** | 0.434(0.233) | -0.015(0.016) | 0.007(0.024) | 0.428(0.075)*** | 0.033(0.098) |
|  |  |  |  |  |  |  |
| Education |  |  |  |  |  |  |
| Diploma/graduate/post graduate*2018-19 | Reference | Reference | Reference | Reference | Reference | Reference |
| Illiterate (including less than primary) *2018-19 | -0.469(0.382) | -0.103(0.377) | -0.074(0.048) | 0.005(0.005) | -0.996(0.247)*** | 0.009(0.179) |
| Primary to higher secondary*2018-19 | -0.764(0.382)** | -0.360(0.361) | -0.096(0.048)*** | -0.016(0.047) | -0.522(0.247)** | 0.033(0.168) |
| **Constant** | **8.106(0.070)***** | **6.975(2.367)***** | **0.885(0.0089)***** | **0.549(0.213)** | **2.855(0.040)***** | **6.661(0.880)***** |
| **Observations** | 53640 | 53622 | 52159 | 52141 | 48427 | 48410 |
| **R^2^** | 0.0170 | 0.211 | 0.024 | 0.352 | 0.012 | 0.575 |
|  |  |  |  |  |  |  |
| **Regular workers** | | | | | | |
| **Time** |  |  |  |  |  |  |
| Year 2011-12 | Reference | Reference | Reference | Reference | Reference | Reference |
| Year 2018-19 | **1.669(0.062)***** | **1.069(0.159)***** | **0.156(0.007)***** | **0.113(0.019)***** | **0.233(0.031)**** | **0.023(0.102)** |
| **Caste groups** |  |  |  |  |  |  |
| Other caste groups | Reference | Reference | Reference | Reference | Reference | Reference |
| Schedule tribe *2018-19 | **0.998(0.303)***** | 0.656(0.628) | 0.048(0.036) | 0.033(0.070) | 0.087(0.144)0.098(0.242) | 0.029(0.222) |
| Schedule caste*2018-19 | 0.828(0.179)*** | 0.524(0.341) | 0.052(0.021)** | 0.034(0.036) | 0.394(0.089)*** | 0.143(0.153) |
| Backward caste *2018-19 | 0.006(0.140) | -0.163(0.237) | 0.004(0.017) | 0.005(0.026) | **0.139(0.069)**** | -0.249(0.112)** |
|  |  |  |  |  |  |  |
| Education |  |  |  |  |  |  |
| Diploma/graduate/post graduate*2018-19 | Reference | Reference | Reference | Reference | Reference | Reference |
| Illiterate (including less than primary) *2018-19 | 1.912(0.196)*** | 1.974(0.615)*** | 0.172(0.024)*** | 0.18(0.06)*** | 0.087(0.098) | 0.242(0.288) |
| Primary to higher secondary*2018-19 | 0.307(0.131)*** | 0.305(0.155)** | -0.003(0.016) | 0.009(0.017) | -0.033(.066) | -0.009(0.107) |
| **Constant** | **4.541(0.071)***** | **4.145(2.881)** | **0.531(0.009)***** | **0.275(0.312)** | **1.686(0.033)***** | **5.497(1.241)***** |
| **Observations** | 70,683 | 70,659 | 67256 | 67233 | 62263 | 62239 |
| **R^2^** | 0.024 | 0.184 | 0.026 | 0.196 | 0.011 | 0.26 |

***p values less than 0.01 and **p value less than 0.05

- Adjusted coefficient (after adjusting all the SES indicators); unadjusted coefficient (without adjusting other SES indicators)

- The regression analysis controls for the following factors: age, house hold type, education, sex, religion, states; clustered at village level. For the comprehensive presentation of results the estimates for these variables are not presented in this table.

-The interaction variables (Caste *2018-19) were created using a dummy variable and then incorporated in the analysis.
